# Serum-derived three-circRNA signature as a diagnostic biomarker for hepatocellular carcinoma

**DOI:** 10.1186/s12935-020-01302-y

**Published:** 2020-06-10

**Authors:** Xiang-Hong Sun, Yu-Tong Wang, Guo-Fu Li, Nan Zhang, Ling Fan

**Affiliations:** 1 Department of Critical Care Medicine, Shengjing Hospital of China Medical University, Shenyang, People’s Republic of China; 2 Department of Clinical Oncology, Shengjing Hospital of China Medical University, Shenyang, People’s Republic of China; 3 Department of Nursing, Shengjing Hospital of China Medical University, Shenyang, People’s Republic of China

**Keywords:** Hepatocellular carcinoma, circRNA, Exosome, Biomarkers, Diagnosis

## Abstract

**Background:**

Hepatocellular carcinoma (HCC) is a common tumor characterized by high morbidity and mortality rates. The importance of circRNA in cancer diagnosis has been established. The study aimed to identify differentially-expressed circRNAs (DECs) in human blood exosomes from patients with HCC and to investigate their diagnostic value.

**Methods:**

The circRNA expression profiles of HCC and normal human blood samples were downloaded and processed from the exoRBase database. At the cutoff criteria of a fold change (FC) > 2.0 and P < 0.05, DECs were screened utilizing the limma package in the R software. A receiver operator characteristic curve (ROC) was used to study its diagnostic value. Quantitative reverse transcription-polymerase chain reaction (qRT-PCR) analysis was performed to confirm the three-circRNAs expression in the blood samples with HCC. Various bioinformatics tools were used to characterize the potential biological pathways induced by circRNAs.

**Results:**

Compared with the normal samples, seven up-regulated and five down-regulated circRNAs were determined in the HCC samples. ROC analyses demonstrated that hsa_circ_0004001, hsa_circ_0004123, hsa_circ_0075792, and a combination of the three biomarkers exhibited higher sensitivity and specificity. The qRT-PCR confirmed that the three circRNAs were upregulated in the blood samples with HCC. Chi squared tests implied that the expression of three circRNAs was positively correlated with the TNM stage and tumor size. The circRNAs participated in VEGF/VEGFR, PI3K/Akt, mTOR, and Wnt signaling pathways by targeting miRNAs.

**Conclusions:**

The study established the existence of seven up-regulated and five down-regulated circRNAs in HCC. Additionally, hsa_circ_0004001, hsa_circ_0004123, hsa_circ_0075792, and a combination of the three were utilized as valuable diagnostic biomarkers in HCC.

## Background

Hepatocellular carcinoma (HCC) is one of the most common malignant gastrointestinal tumors worldwide and is characterized by high morbidity and mortality rates [1]. In China, annual HCC mortality rate ranks second in the total tumor mortality rates. It is noteworthy that the onset of the disease is often hidden and the condition progresses rapidly [2]. Unfortunately, upon diagnosis, the majority of the patients are in advanced stages of the disease. Thus, early diagnosis is key in improving the HCC treatment and prognosis. Liver cancer screening in the clinic is typically based on the detection of serum alpha-fetoprotein and various imaging methods. However, the sensitivity and specificity of the serum alpha-fetoprotein (AFP) detection are limited. Moreover, imaging techniques are costly and the patients’ compliance is poor.

The circRNA is a type of non-coding RNA, which is widely present in many organisms, and exhibits a covalent closed loop structure [3]. Studies have confirmed that circRNA is abundantly expressed, stable, and conservative, and displays specificity in different species, diseases, tissues, and developmental stages. Considering the unique molecular biological characteristics, circRNA secreted into human circulating blood can be used as a biomarker for disease diagnosis [4]. In recent years, a number of studies have indicated that circRNA plays vital roles in tumorigenesis and progression of HCC, and could be used as a diagnostic biomarker. For example, hsa_circ_0001445 has been shown to inhibit the proliferation, migration, and invasion of HCC cells. It also promotes apoptosis, and thus, serves as a useful diagnostic biomarker [5]. Nonetheless, the role of multiple circRNAs in the diagnosis of patients with HCC remains largely unknown.

In the present study, we identified a number of differentially expressed circRNAs (DECs) in blood with HCC. The increased expression levels of hsa_circ_0004001, hsa_circ_0004123, and hsa_circ_0075792 were verified utilizing quantitative reverse transcription-polymerase chain reaction (qRT-PCR) analysis. The expression of the three circRNAs was positively correlated with the TNM stage and tumor size. In addition, the combination of hsa_circ_0004001, hsa_circ_0004123, and hsa_circ_0075792 can be used as a new biomarker for the diagnosis of patients with HCC.

## Methods

### The circRNA expression data

The circRNA expression profiles, which included 21 HCC and 32 normal blood samples, were obtained from the exoRBase (http://www.exorbase.org/exoRBase/toIndex) database [6]. The expression matrix was preprocessed using the following exclusion criteria: (1) circRNA names were not integrated; and (2) circRNAs with an expression level of “0” were more than half, with 53 samples in total. Finally, a total of 857 circRNAs, which had proper expression data, were included for further analysis.

### Identification of DECs

The 32 healthy blood samples were tested as the normal group. At the cutoff criteria of P < 0.05 and fold-change (FC) > 2.0, the DECs in 21 HCC blood samples were identified utilizing the limma package in the R software.

### Serum samples

The study enrolled 71 patients diagnosed with HCC at Shengjing Hospital of the China Medical University. Forty healthy individuals were enrolled as controls. Fasting venous blood samples were collected, and none of the patients received any treatment prior to collection. Ethylenediamine tetraacetic acid was added for anticoagulation, and all samples were centrifuged at 1600×*g* for 10 min at 4 °C. Following high-speed centrifugation at 12,000×*g* for 10 min, the plasma was separated and stored at – 80 °C until needed. The present study was conducted with the approval of the ethics review committee of the Shengjing Hospital of China Medical University (No.: 2018PS362K), and all of the research participants signed informed consent form.

### RNA isolation and quantitative reverse transcription-polymerase chain reaction (qRT-PCR)

Eight hundred μL of the TRIzol^®^ reagent (Invitrogen, Carlsbad, CA, USA) was added to each 200 μL blood sample, and the extraction procedure described in the manufacturer’s instructions was followed. The nucleic acid OD260/OD280 ratio was determined using a NanoDrop spectrophotometer (Thermo Fisher Scientific, Waltham, MA, USA). The values obtained for the samples were in the range of 1.8–2.0. The synthesis of cDNA and the qRT-PCR reactions were conducted using a reverse transcription kit, as well as the SYBR fluorescence quantitative kit (TaKaRa, Shiga, Japan). The reaction system was 20 μL in volume and consisted of 10 μL of SYBR Premix Ex Taq, 0.5 μL of cDNA, 0.5 μL of upstream and downstream primers, 0.5 μL of ROX, and 8 μL of ddH_2_O. The primers for target circRNAs and the internal reference GAPDH are listed in Table 1. All steps were conducted according to the manufacturer’s instructions. The relative gene expression was quantified using the 2^−∆∆ct^ method.

**Table 1:** The primer sequences for qRT-PCR

Names	Sequence (5′–3′)
hsa_circ_0004001	
Forward	ACGAACATCACAGTACATTGGT
Reverse	GGCTTTAAGTCTGTGTGAGTCA
hsa_circ_0004123	
Forward	CTGTCTCCCCGCCTGAAG
Reverse	TGGTGCACATTATCCACGGA
hsa_circ_0075792	
Forward	GTGTTCTATGACATGGATCCCC
Reverse	TCTTCTCTGACAACGGTGGA
GAPDH	
Forward	AGCCACATCGCTCAGACAC
Reverse	GCCCAATACGACCAAATCC

### Target miRNAs prediction of circRNA and functional enrichment analysis

To further explore the molecular mechanism of circRNAs, the online Circular RNA Interactome [7] tool was used to predict the downstream targeted regulatory miRNAs of hsa_circ_0004001, hsa_circ_0004123, and hsa_circ_0075792. FunRich v2.0 (http://funrich.org) [8], which is a common gene biological pathways enrichment analysis software, was used for investigating the potential signaling pathways of targeted miRNAs.

### Statistical analysis

The paired and unpaired t-tests were conducted for group comparisons. The comparisons of gene expression and patient clinical features were conducted by Chi square test. The receiver operating characteristic (ROC) curve and the corresponding area under the curve (AUC) were determined for individual and combined circRNAs. All statistical analyses were performed using SPSS version 20.0 (SPSS Inc., Chicago, IL, USA), and the data were considered statistically significant when P < 0.05.

## Results

### DECs in serum of HCC patients

**Fig. 1:**
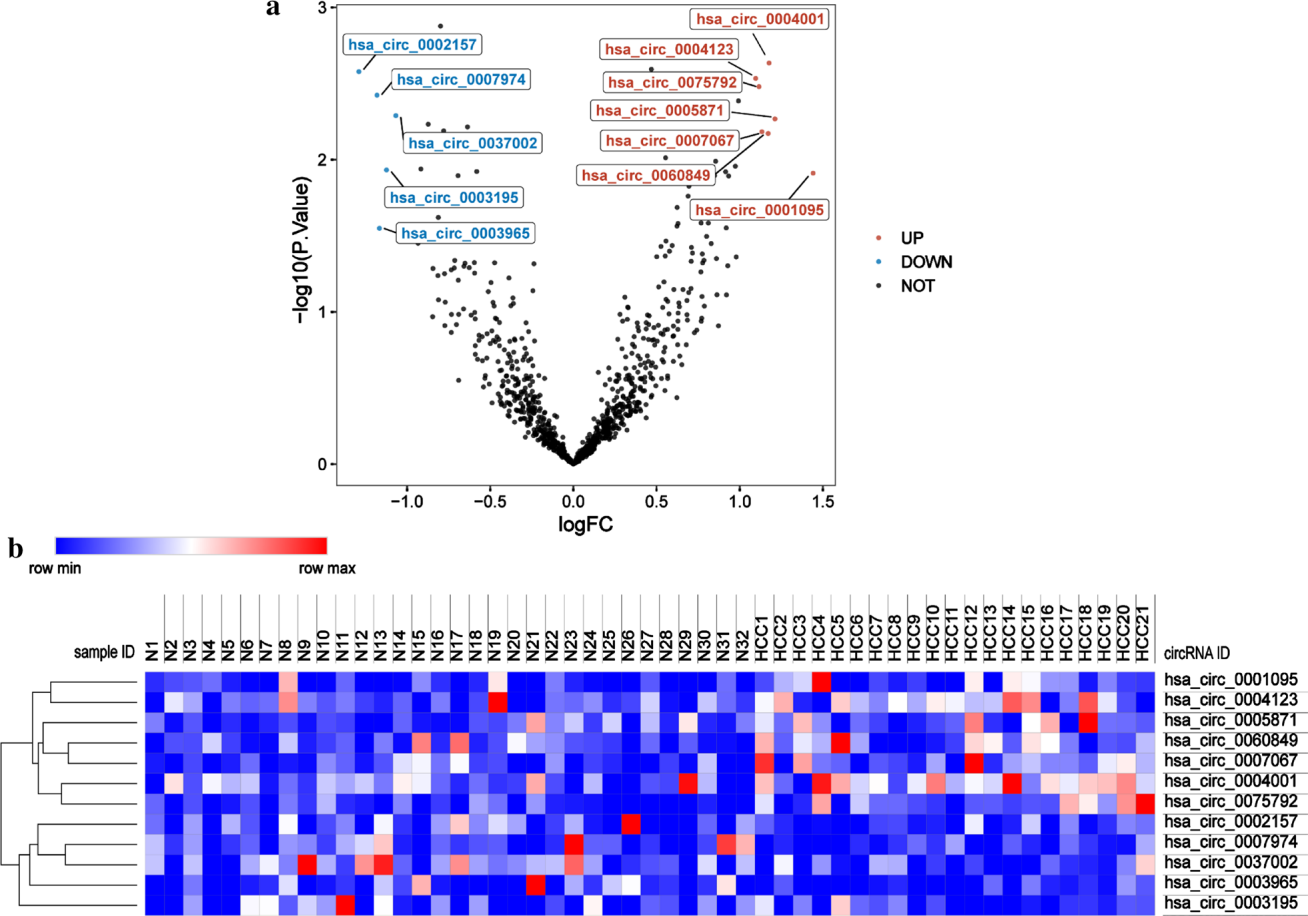
The visualization of DECs in HCC and normal samples. **a** Seven up-regulated and five down-regulated circRNAs were identified in HCC. **b** Hierarchical clustering analysis of the DECs in 21 HCC serum and 32 normal samples

**Fig. 2:**
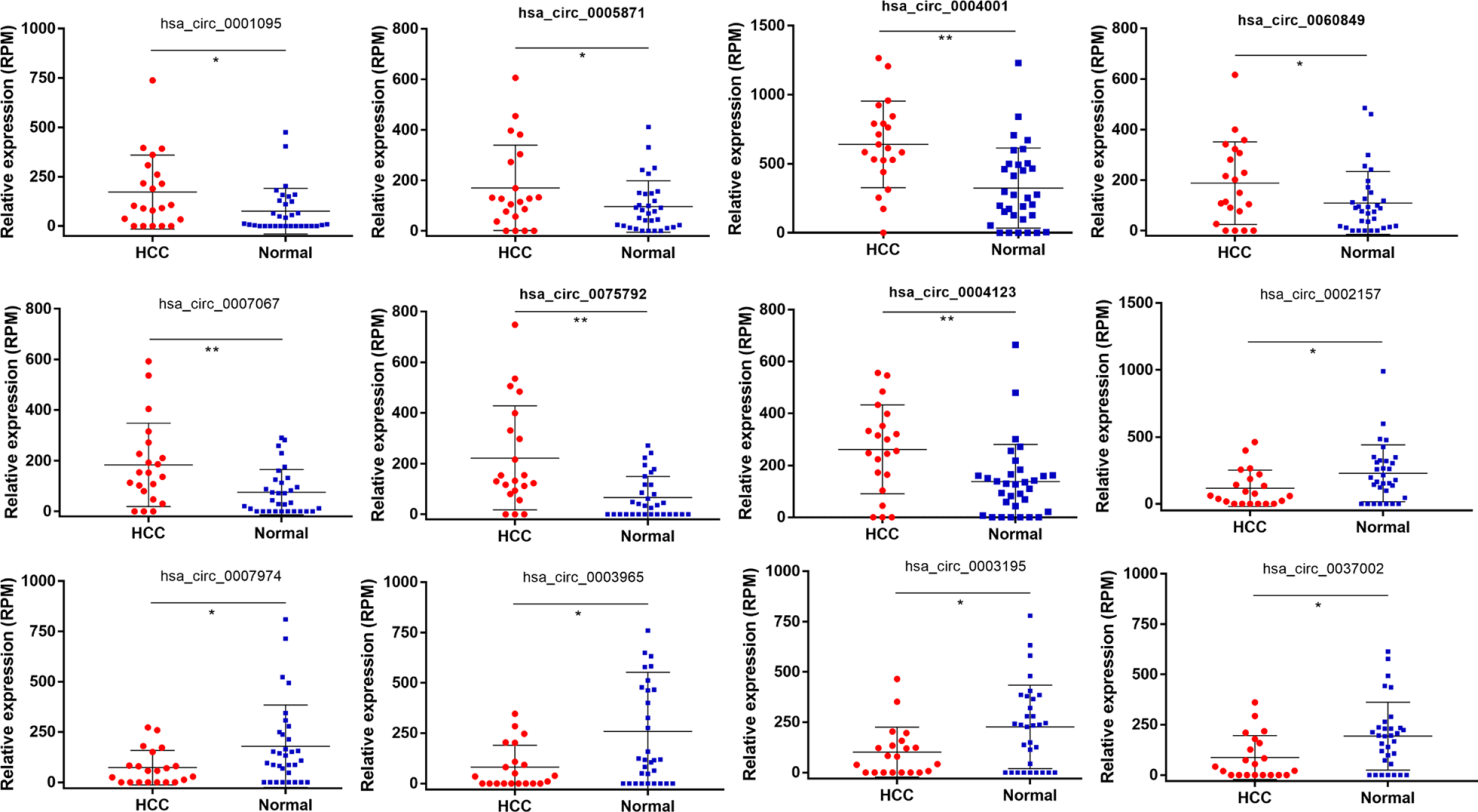
The relative expression level of DECs in HCC serum and normal samples

Compared with normal samples, seven up-regulated and five down-regulated circRNAs were detected in the HCC blood samples. The DECs and the corresponding FC and p-values are listed in Table 2. A volcano plot was used for visualizing the DECs (Fig. 1a). In addition, the heat map of hierarchic cluster analysis indicated that the DECs could be discriminated between HCC and normal blood samples (Fig. 1b). The expression levels of 12 DECs in HCC and normal samples are shown in Fig. 2.

**Table 2:** The differentially expressed circRNAs in the serum of HCC patients

circRNAs	Up-regulated		circRNAs	Down-regulated	
	logFC	P value		logFC	P value
hsa_circ_0001095	1.44	0.012	hsa_circ_0002157	− 1.29	0.003
hsa_circ_0005871	1.21	0.005	hsa_circ_0007974	− 1.18	0.004
hsa_circ_0004001	1.18	0.002	hsa_circ_0003965	− 1.17	0.028
hsa_circ_0060849	1.17	0.007	hsa_circ_0003195	− 1.12	0.012
hsa_circ_0007067	1.13	0.007	hsa_circ_0037002	− 1.07	0.005
hsa_circ_0075792	1.12	0.003			
hsa_circ_0004123	1.10	0.003

### The diagnostic significance of circRNA in HCC

**Fig. 3:**
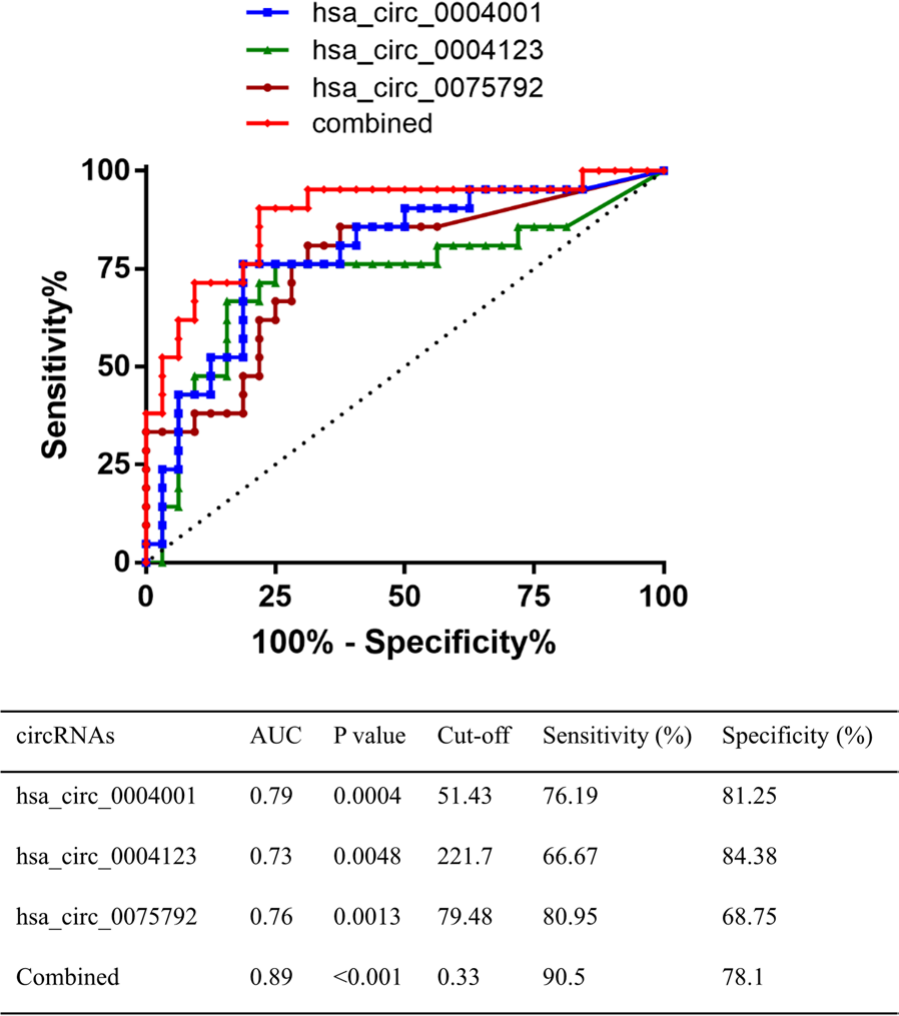
Diagnostic value of three-circRNAs. The ROC, sensitivity, specificity, and AUC values are shown

ROC curves were used to explore the diagnostic value of the differentially expressed circRNAs. It was found that hsa_circ_0004001, hsa_circ_0004123, and hsa_circ_0075792 exhibited adequate sensitivity and specificity in discriminating HCC patients from the healthy subjects. Furthermore, the combined three-circRNA (hsa_circ_0004001, hsa_circ_0004123, and hsa_circ_0075792) considerably improved the sensitivity, specificity, and AUC values for diagnosis (Fig. 3). These results suggested that the three circRNA signature could be utilized as a diagnostic biomarker in HCC.

### The qRT-PCR expression validation and analysis of the clinical feature relationships

**Fig. 4:**
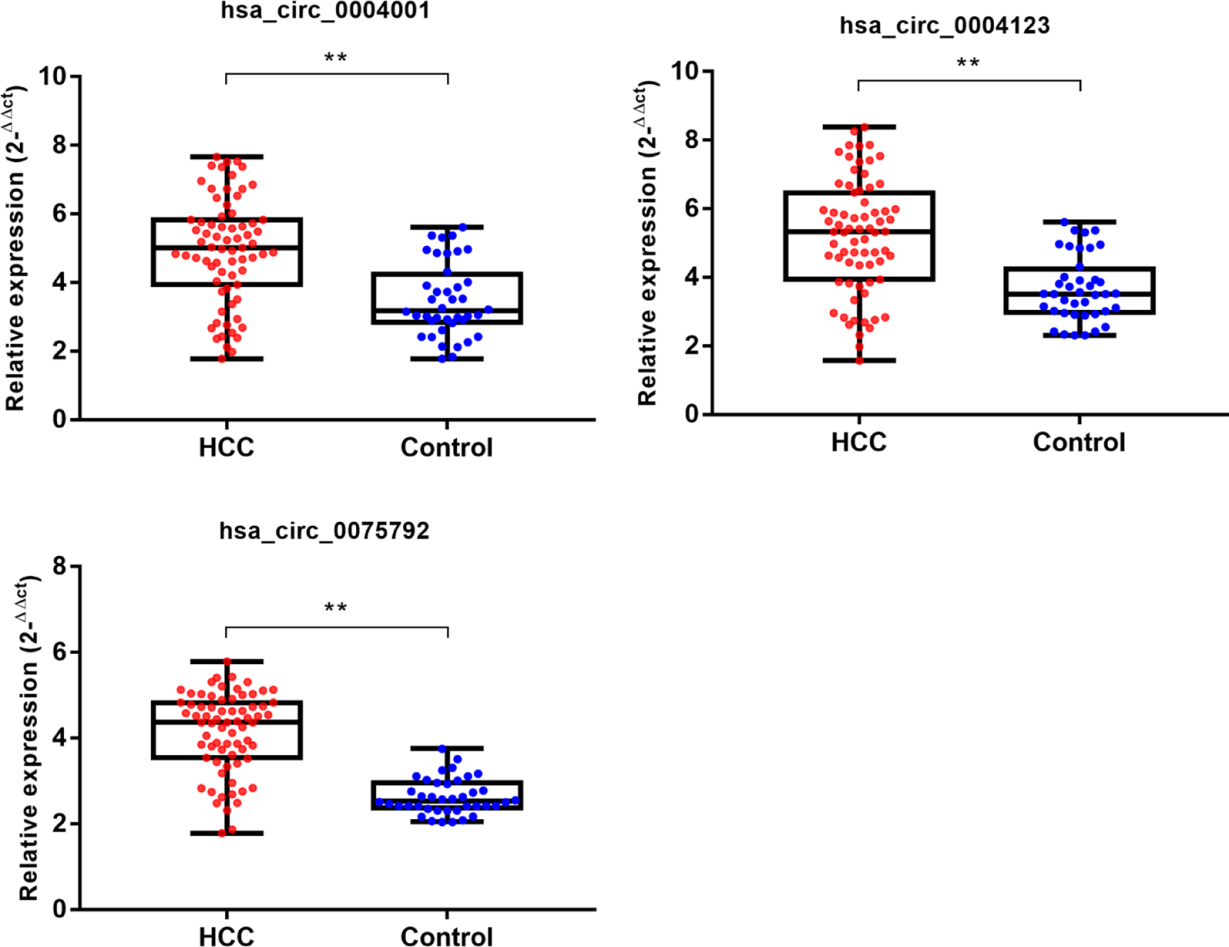
The qRT-PCR assay validation for three-circRNAs expression. Compared with the normal samples, hsa_circ_0004001, hsa_circ_0004123, and hsa_circ_0075792 were all notably up-regulated in the HCC serum samples

The expression levels of hsa_circ_0004001, hsa_circ_0004123, and hsa_circ_0075792 in the serum samples of HCC patients and healthy volunteers were analyzed by qRT-PCR. The results suggested that the expression levels of hsa_circ_0004001, hsa_circ_0004123, and hsa_circ_0075792 in the HCC patient blood samples were considerably higher than those in the samples of healthy individuals (Fig. 4). The relationships between the circRNAs expression levels and clinicopathological features of the HCC patients were examined using the Chi square test. The results showed that the three circRNA expression levels were positively correlated with the TNM stage and tumor size. However, age, sex, and lymph node metastasis were not significantly correlated (Table 3).

**Table 3:** Relationship between circRNAs expression and clinical features of HCC patients

Variable	hsa_circ_0004001 expression		*P* value	hsa_circ_0004123 expression		*P*-value	hsa_circ_0075792 expression		*P*-value
	Low	High		Low	High		Low	High	
Age									
< 60	19	15	0.542	20	14	0.278	18	16	0.414
≥ 60	18	19		17	20		16	21	
Sex									
Male	17	17	0.909	18	16	0.764	19	15	0.941
Female	18	19		18	19		21	16	
TNM staging									
I–II	20	14	0.014*	19	15	0.702	22	12	0.024*
III–IV	11	26		19	18		14	23	
Tumor size									
< 5 cm	15	19	0.043*	18	16	0.006*	22	12	0.112
≥ 5 cm	8	29		8	29		17	20	
Lymph node metastasis									
Negative	17	17	0.205	17	17	0.424	14	20	0.86
Positive	13	24		15	22		16	21	

*P < 0.05 and **P < 0.01, statistically significant

### Targeted miRNA prediction and gene function enrichment analysis

**Fig. 5:**
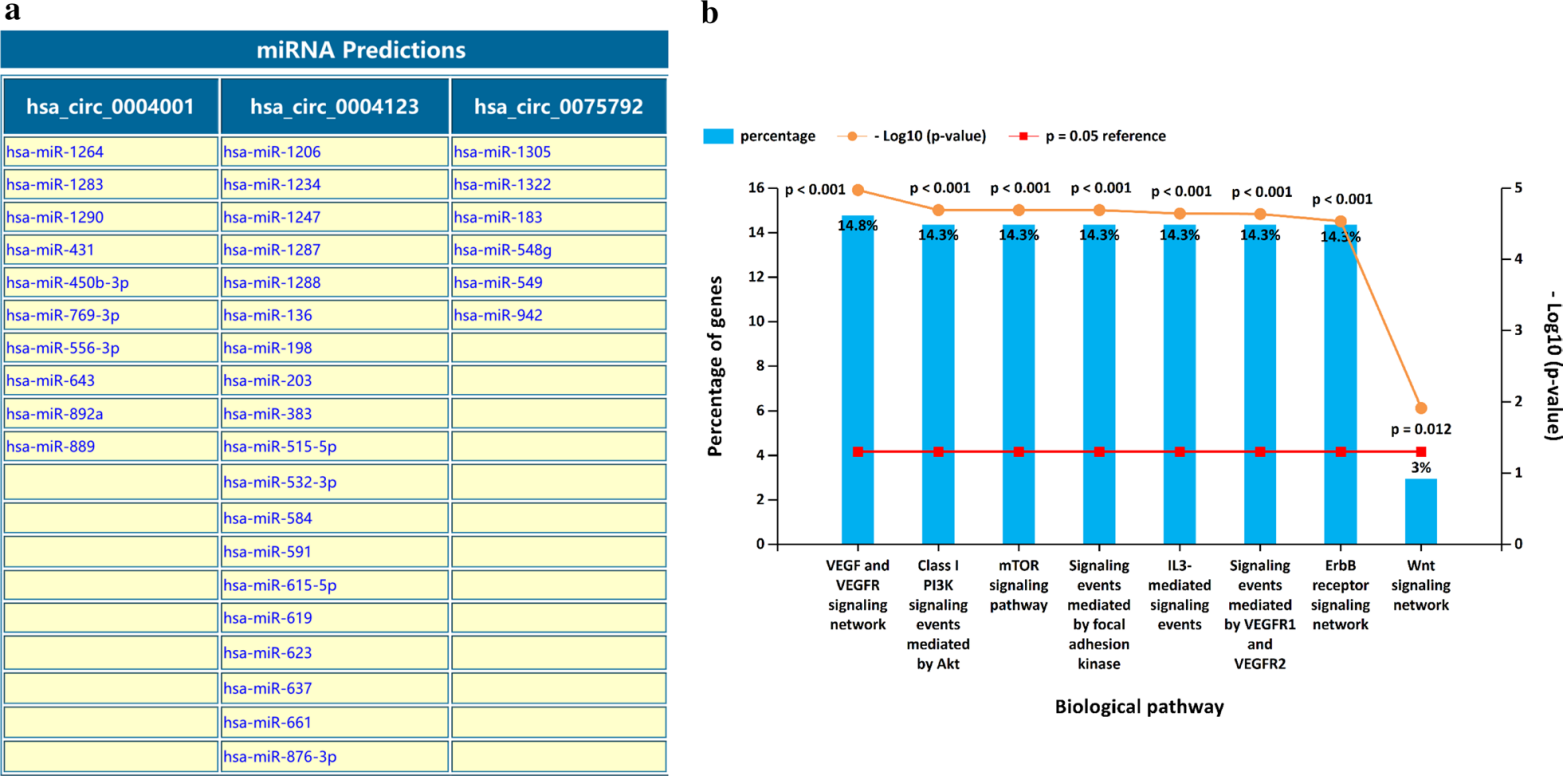
miRNAs prediction and biological pathway analysis related to three-circRNA. **a** There were 10, 19, and 6 miRNAs identified as targets of hsa_circ_0004001, hsa_circ_0004123, and hsa_circ_0075792, respectively. **b** Pathway enrichment analysis indicated that hsa_circ_0004001, hsa_circ_0004123, and hsa_circ_0075792 participated in the VEGF/VEGFR, PI3K/Akt, mTOR, and Wnt signaling pathways

The results obtained from the Circular RNA Interactome evaluation showed that hsa_circ_0004001, hsa_circ_0004123, and hsa_circ_0075792 interacted with 10, 19, and six miRNAs, respectively, by targeting regulation (Fig. 5a). Because different circRNAs can regulate the same miRNA, a total of 35 miRNAs were obtained after excluding duplicate results. Pathway enrichment analysis of these 35 miRNAs utilizing the FunRich tool indicated that hsa_circ_0004001, hsa_circ_0004123, and hsa_circ_0075792 participated in biological signaling pathways, such as VEGF/VEGFR, PI3K/Akt, mTOR, and Wnt (Fig. 5b). Together, the results revealed that hsa_circ_0004001, hsa_circ_0004123, and hsa_circ_0075792 regulated the expression of several miRNAs and were involved in important signaling pathways.

## Discussion

HCC is the most important pathological type of primary liver cancer. It is characterized by strong invasiveness, high malignancy, and poor prognosis. In China, the mortality rate associated with HCC ranks second among malignant tumors of the digestive system, and is second only to gastric cancer [9]. At present, the most effective way to treat liver cancer is to perform immediate radical resection. Thus, the key to improving the available liver cancer treatment is to increase the early diagnosis rate.

CircRNA was first found in plant-like viruses in 1976, when it was considered to be a product of miss-splicing and did not receive any significant attention [10]. With the development of high-throughput sequencing technology in recent years, circRNA has been found to form as a result of human gene transcription. This finding has led to more in-depth studies concerning circRNAs [11]. An increasing number of studies have shown that circRNAs play important roles in numerous human diseases, particularly in the process of tumor development. CircRNAs can function at the level of gene transcription and translation [12, 13]. For example, circRNA competitively attenuates the inhibitory effect of miRNA on downstream target genes by acting as a miRNA “sponge” [14]. Huang et al. found that circRNA-100338 is involved in activating the mTOR signaling pathway through the circRNA-100338/miR-141-3p/RHEB axis to promote HCC cell proliferation [15]. Moreover, in breast cancer, circRNA_002502 regulates the sensitivity and tumor progression of the antitumor drug, tamoxifen, via the miR-182-5p/FOXO3a axis [16]. These outcomes confirmed the crucial role of circRNA in tumorigenesis and development. In addition, multiple studies have determined that circRNA can be utilized as a diagnostic marker for tumors. Wei et al. found that circRNA_102958 was significantly upregulated in gastric cancer, and its AUC value was 0.74, making it a useful molecular marker for gastric cancer diagnosis [17]. Moreover, circRNA hsa_circ_0091579 has previously been established as a biomarker for the diagnosis and prognosis of HCC [18].

In the present study, we downloaded the RNA-Seq data for circRNA from HCC blood samples in the exoRBase database. The exoRBase database stores RNA-Seq of circRNA, lncRNA, and mRNA in blood exosomes from various human diseases, as well as published experimental data [19]. The database stores 21 HCC blood samples and 32 normal human blood samples. The sample size and the types of circRNAs sequenced are large, providing strong evidence for the results of our study. Following screening, seven circRNAs with up-regulated expression and five circRNAs with down-regulated expression were found in the HCC blood samples.

To further explore the diagnostic role of up-regulated circRNA in HCC, we conducted ROC curve analysis. The results demonstrated that hsa_circ_0004001, hsa_circ_0004123, and hsa_circ_0075792 exhibited higher diagnostic sensitivity and specificity, and the AUC values of all three were greater than 0.7. The qRT-PCR verification experiments also showed that hsa_circ_0004001, hsa_circ_0004123, and hsa_circ_0075792 were significantly higher in the plasma of liver cancer patients, when compared with those of the healthy group. At present, serum AFP is the preferred diagnostic marker for primary liver cancer. However, various studies have shown that there is a possibility of false negative diagnosis using serum AFP; thus, this technique is characterized by poor diagnostic sensitivity and specificity [20]. Previous research has found that has_circ_0000745 combined with carcinoembryonic antigen detection is more accurate in the early diagnosis of gastric cancer [21]. This implies that the combined detection of multiple biomarkers has great advantages in tumor diagnosis. Consequently, the present study further investigated the role of the above three circRNAs in the diagnosis of hepatocellular carcinoma. We found that the combination of the three circRNAs notably improved the diagnostic sensitivity and specificity of a single marker, with an AUC value of 0.885. Although numerous studies have described the importance of circRNA in the diagnosis of HCC, the majority of the approaches involve single-markers [22, 23]. Conversely, we screened and identified three circRNAs as combined markers with high diagnostic sensitivity and specificity.

Further exploration of the downstream regulatory mechanism of circRNA revealed that hsa_circ_0004001, hsa_circ_0004123, and hsa_circ_0075792 regulated 35 target miRNAs, and participated in key biological signaling pathways, such as VEGF/VEGFR, PI3K/Akt, mTOR, and Wnt. Zhang et al. found that miRNA-146a inhibits distant metastasis of hepatocellular carcinoma by downregulating VEGF expression through multiple pathways [24]. Furthermore, recent research showed that elongation factor 2 kinase promotes angiogenesis in HCC through the PI3K/Akt signaling pathway [25]. However, Ma et al. confirmed that silencing lncRNA HEIH inhibited liver cancer cell proliferation and metastasis through the miR-199a-3p/mTOR axis [26]. The above observations imply that hsa_circ_0004001, hsa_circ_0004123, and hsa_circ_0075792 possibly affect the occurrence and progression of HCC through a variety of tumor-related signaling pathways, providing a theoretical basis and research direction for further exploration of its mechanism of action. However, there are also some limitations in the study design. The present study only comfirmed the expression level of the three circRNAs in HCC and explored their potential molecular function in silico. In the future study, we would investigate the accurate biological roles of circRNAs in vitro and in vivo to obtain more conclusive evidences.

## Conclusions

The present study identified a number of DECs in the HCC blood samples, which provided a valuable platform for further exploration of its molecular mechanism of action. In addition, the combination of hsa_circ_0004001, hsa_circ_0004123, and hsa_circ_0075792 is a promising biomarker for the diagnosis of patients with HCC.

## Data Availability

The datasets used and/or analysed during the current study are available from the corresponding author on reasonable request.

## Abbreviations

HCC: Hepatocellular carcinoma
DECs: Differentially-expressed circRNAs
FC: Fold change
ROC: Receiver operator characteristic curve
qRT-PCR: Quantitative reverse transcription-polymerase chain reaction
AFP: Alpha-fetoprotein
AUC: Area under the curve
